# Systematic Review of Yoga for Pregnant Women: Current Status and Future Directions

**DOI:** 10.1155/2012/715942

**Published:** 2012-08-14

**Authors:** Kathryn Curtis, Aliza Weinrib, Joel Katz

**Affiliations:** Department of Psychology, Faculty of Health, York University, Toronto, ON, Canada M3J 1P3

## Abstract

*Objectives*. Yoga is used for a variety of immunological, neuromuscular, psychological, and pain conditions. Recent studies indicate that it may be effective in improving pregnancy, labour, and birth outcomes. The purpose of this paper is to evaluate the existing literature on yoga for pregnancy. *Methods*. Six databases were searched using the terms “yoga AND pregnancy” and “yoga AND [post-natal OR post-partum]”. Trials were considered if they were controlled and evaluated a yoga intervention. All studies were evaluated for methodological quality according to the Jadad scale and the Delphi List. *Results*. Six trials were identified: three were randomized controlled trials (RCTs) and three were controlled trials (CTs). The methodological quality and reporting ranged from 0–5 on the Jadad scale and from 3–6 on the Delphi List. Findings from the RCT studies indicate that yoga may produce improvements in stress levels, quality of life, aspects of interpersonal relating, autonomic nervous system functioning, and labour parameters such as comfort, pain, and duration. *Conclusions*. The findings suggest that yoga is well indicated for pregnant women and leads to improvements on a variety of pregnancy, labour, and birth outcomes. However, RCTs are needed to provide more information regarding the utility of yoga interventions for pregnancy.

## 1. Introduction

Yoga is an ancient mind-body practice that originated in India and is becoming increasingly recognized and used in developed nations as a health practice for a variety of immunological, neuromuscular, psychological, and pain conditions [1, 2]. The word yoga comes from the Sanskrit term “yug” and directly translates as “to unite”; more broadly, it means to work towards a unified experience of the self and improved health [3]. Most recognized for its potential to create balance along emotional, mental, physical, and spiritual dimensions, yoga is a comprehensive system that uses physical postures (*asana*), breathing exercises (*pranayama*), concentration and meditation (*dharana* and *dhyana*), and contemplative practice. Although there are a plethora of lineages and schools of yoga that are offered in modern society, practices typically include at least the physical postures and breathing exercises. Yoga is thought to alter nervous system regulation and physiological system functioning (e.g., immune, endocrine, neurotransmitter, and cardiovascular) and improve psychological well being (e.g., frequency of positive mood states and optimism) and physical fitness (e.g., strength, flexibility, and endurance) [2].

Pregnancy is a condition in which women undergo distinct physiological changes and stress and is accompanied by unique physical and psychological demands. There is a need to manage the various physical, emotional, mental, and pain states that arise throughout the stages of pregnancy and labour. The well being and quality of life of the mother is critical for optimal pregnancy outcomes; self-soothing techniques, psychoeducation, and relaxation are particularly important in this transitional and meaningful time [4]. Maternal stress and anxiety during pregnancy is associated with a host of negative consequences for the fetus and subsequent development. For instance, fetal exposure to maternal stress and stress-related peptides is a risk factor for adverse outcomes on the programming of the nervous system and brain morphology of fetuses, infants, and children. Early gestational stress exposure is associated with negative outcomes at different developmental stages, slowed maturation and behavioural response patterns in fetuses, alterations in neonatal stress regulation and behavioural reactions to stress, blunted cognitive functions and emotional and behavioural problems in infants and toddlers, and reduced brain volume in areas associated with cognitive function in children [5]. In addition, prenatal maternal stress and anxiety may be risk factors for potential negative consequences for children later in life, such as the development of attention deficit hyperactivity disorder or lowered performance on aspects of executive function [6, 7]. It is hypothesized that maternal stress may affect the intrauterine environment and alter fetal development during critical periods, through either activation of the placental stress system, causing the release and circulation of corticotropin releasing hormone, or through diminished blood flow and oxygen to the uterus [8]. Therefore, it is important to regulate maternal stress and provide expecting mothers with coping strategies for the inevitable stresses and changes that occur during pregnancy to increase quality of life and to maximize infant health and development.

Physical exercise can be helpful in the management of stress and other associated conditions or symptoms accompanying pregnancy, such as edema, gestational hypertension or diabetes, mood instability, musculoskeletal discomfort, aches, and weight gain [9]. Engaging in physical exercise during pregnancy was once regarded as a risky behaviour, although it is increasingly recognized as safe and is encouraged in routine prenatal care. Melzer et al. [9] concluded that regular physical exercise has maternal and fetal advantages that outweigh risks and recommend at least 30 minutes of exercise, most days of the week for the prevention and treatment of conditions associated with inactivity, such as gestational diabetes and hypertension.

Mind-body practices that cultivate general health, diminish distress, and increase self awareness, such as tai chi or yoga, maybe be particularly effective in addressing both the physical and psychoemotional aspects of pregnancy and labour [4]. Other related practices, including biofeedback, meditation, and imagery, have been found to reduce anxiety and endocrine measures, such as cortisol, in women during labour [10, 11]. Relaxation therapies for pain management in labour have also become popular as women are seeking alternatives to traditional treatment approaches, including analgesics and anesthesia, which can be invasive and are sometimes associated with negative side effects for both the mother and infant [12].

Labour pain is a subjective and multidimensional experience that varies according to each woman's individual perceptions of and reactions to nociceptive information during labour and is influenced by psychosocial, cognitive, and physiological factors [13]. It is suggested that practitioners use a multidisciplinary approach to pain management in labour and incorporate both pharmacological and nonpharmacological approaches that can be tailored to individual preferences and needs [14]. Confidence, self-efficacy, and coping ability are considered important for a positive labour experience, and maternal prenatal anxiety is negatively associated with prelabour self-efficacy for child-birth and labour pain [15]. Other psychological factors, such as pain catastrophizing, have been associated with greater lumbopelvic pain during pregnancy and with decreased postpartum physical ability [16] and can also predict the request for pain relief during labour [17].

Yoga may be effective in the reduction of negative symptoms associated with pregnancy and birth. Given that 35% of women aged 28–33 years already practice yoga, it is important to evaluate its effects on the maternal experience of stress, anxiety, pain, discomfort, and other variables as well as on labour and birth outcomes [18]. A recent review of yoga for pregnancy related outcomes concluded that yoga is positively indicated for use in pregnancy but the findings are not definitive since some of the trials included in that review were uncontrolled and others demonstrated poor methodological quality for different reasons [19]. The primary purpose of the present paper is to systematically evaluate the evidence for the use of yoga during pregnancy and labour and to make recommendations for the direction of future research.

## 2. Materials and Methods

Literature searches were conducted to identify all controlled clinical trials of yoga and pregnancy. The following databases were used: EBSCOHost Web: CINAHL, Pubmed, Medline, Proquest, PsychoInfo and “Evidence Based Medicine Reviews: Cochrane DSR, ACP Journal Club, DARE, and CCTR”. The two terms “yoga” and “pregnancy” were linked together using the Boolean operator “AND” in order to search articles containing both terms. In addition, a search containing the terms “yoga AND [post-natal OR post-partum]” was conducted. The reference lists of located articles were also searched for possible publications. Only articles in English were included.

Yoga was defined as a mind-body practice that included traditional physical postures and may incorporate other components, such as breathing exercises and meditation. Only studies that used yoga postures explicitly as an intervention were included; interventions that employed other aspects of yoga, such as yogic breath, yogic philosophy, ayurvedic herbs, or mindfulness as the primary intervention, were not included, as the effects of asana or integrated yoga programs were of primary interest. Information on trial design, randomization, blinding, drop out rate, inclusion and exclusion criteria, details about treatment and control conditions, main outcome measures, and main results were extracted, as has been done in previous reviews of yoga for certain conditions [20].

Studies were evaluated independently by two reviewers (K. Curtis and J. Katz) according to the five-item Jadad scale [21] and the nine-item Delphi List [22]; any differences were resolved through discussion until a consensus was reached. The Jadad scale evaluates studies according to a five-item rating scale, with each item awarded one point for a “yes” answer and zero points for a “no” answer. By selecting a commonly used rating scale, the findings of the present paper are more easily compared with review articles that evaluate related interventions. The items include the following questions: (1) was the study randomized, (2) was randomization explained and appropriate, (3) was the study double blinded, (4) was the process of double blinding explained and appropriate, and (5) was information provided on the number and reasons for participant drop out or withdrawal. Points are deducted if randomization or blinding is inappropriate. Double-blinding is intrinsically challenging in trials involving a yoga intervention, so a modified approach to questions 3 and 4 was used, in which a point was awarded to studies that used a single blind approach, and a second point was awarded if the authors explained how the assessor/statistician was blinded.

The Delphi List more specifically addresses the issue of blinding and also includes items concerning other important aspects of clinical trials. It contains two separate items for care provider blinding and patient blinding, making it amenable to evaluating trials in which participant blinding is impossible. This nine item scale includes a series of questions which are rated according to yes/no/don't know, with one point awarded for ratings of “yes” answers and zero points awarded for “no” or “don't know”. The items include: (1) treatment allocation: (a) was a method of randomization performed and (b) was the treatment allocation concealed, (2) were the groups similar at baseline regarding the most important prognostic indicators, (3) were the eligibility criteria specified, (4) was the outcome assessor blinded, (5) was the care provider blinded, (6) was the patient blinded, (7) were point estimates and measures of variability presented for the primary outcome measures and (8) did the analysis include an intention-to-treat analysis. Finally, given the limited number of studies published on yoga for pregnancy, in addition to the varying designs methodology of the studies, a meta-analysis was not performed and the studies are presented descriptively.

## 3. Results 

**Figure 1:**
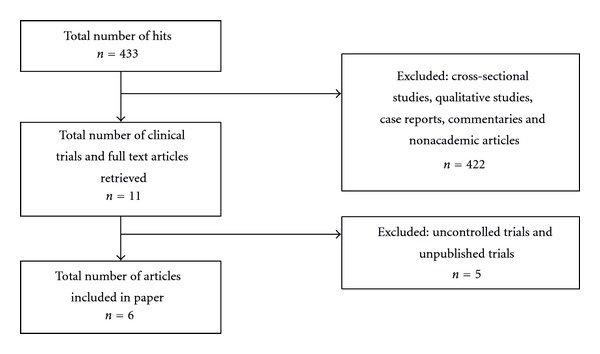
Flow diagram of articles.

The search strategy, “yoga AND pregnancy” and “yoga AND [post-natal OR post-partum]”, generated 433 initial results (Figure 1), including nonacademic results (EBSCOHost Web; 72, Pubmed; 49, Medline; 41, Proquest; 251, PsychInfo; 17, Cochrane; 3). We excluded cross-sectional surveys, case reports, qualitative studies, and commentaries. Of the remaining articles, a total of 11 clinical trials were retrieved for further evaluation including three unpublished studies (a presentation at a conference and two doctoral dissertations). Of these 11 trials, we excluded five on the basis that they were pilot studies and did not have a control group (three used the data from the same intervention) [23–27]. Five controlled studies, resulting in six publications, involving 689 participants were eligible and are included in the present paper (Table 1) [28–33].

The six studies originated in India [29–32], Taiwan [28], and Thailand [33]. All studies had participants who were either (1) primigravida [28, 33], (2) primigravida or multigravida with one living child [31, 32], or (3) primigravida or multigravida [29, 30]. Three of the six studies were randomized [31–33] and three were not [28–30]. Exclusion criteria included a variety of medical conditions and complications such as: diabetes, hypertension, obesity, multiple pregnancy, history of previous pregnancy loss due to known single gene defects, chromosomal disorders, intrauterine infections, in vitro fertilization pregnancy, previous history of intra uterine growth retardation, preecclampsia, maternal structural abnormalities, fetal abnormality on ultrasound scanning, multigravida with no living children, psychiatric problems, being younger than 18, abnormal extremities (unable to do activities), unable to speak the native language, previous exposure to yoga, and regular exercise for one year. Furthermore, one study excluded women if they were admitted to hospital during active labour and if they received epidural anesthesia or a caesarean section, in accordance with the methodology for data collection of that study [28]. 

The various components of a yoga practice that were included in the reviewed trials included postures (*asana*), breathing practices (*pranayama*), concentration/meditation (*dharana/dhyana*), deep relaxation or yoga sleep (yoga *nidra*), lectures/counseling sessions on lifestyle change, and information on anatomy and chanting (Table 2). Typically, yoga programs that assimilate multiple aspects of yoga, such as physical postures, breathing practices, and lectures on yogic philosophy, are considered “integrated” yoga programs. Some interventions incorporated teachings from ancient yogic texts, such as Patanjali's yoga sutras [32, 33], while another had a greater emphasis on yoga as exercise [28]. The yoga programs commenced either on week 18–20 of gestation, resulting in a 16–20 week-long intervention [29–32] or at week 26–28 of gestation, resulting in an 10–14 week-long intervention [28, 33]. Yoga interventions consisted of weekly practice of thirty minutes to one hour, three times a week [28, 31–33], or one hour daily [29, 30]. Control interventions included a walking group [29, 30], standard prenatal exercises [31, 32], and routine nursing care [28, 33].

All studies except one [33] provided a list of the postures used in the yoga interventions and four of the studies tailored the interventions to differ across trimester, according to the evolving needs of the pregnant women [29–32]. The two studies that included standard antenatal exercises as a control intervention used the same set of exercises, as approved by the Executive Council of the Society of Obstetrician and Gynecologists of Canada and by the Board of Directors of the Canadian Society for Exercise Physiology and provided them in table format [31, 32]. The standard antenatal exercise condition consisted of lectures (e.g., physiology of pregnancy, modern science concepts of a healthy lifestyle, and management of stress), a series of stretches (e.g., hamstring, thigh, and calf), strengthening exercises (e.g., squatting, push ups, and seated rowing), walking, and supine rest. 

All studies provided participants with materials with which to practice at home, including cassettes, booklets, and videos. Three of the studies monitored participant adherence to home practice with diaries and phone calls [28, 31–33], one study used only phone calls [28] and two did not report that they monitored participant home practice [29, 30]. Most studies used valid and reliable measures of the dependent variables [28, 31–33], two also used researcher modified measures or measures developed for the particular trial [28, 33] and three used primarily quantitative information that was documented at birth or extracted from hospital records [29, 30, 33]. Statistical analysis was overly liberal in terms of the Type I error rate in most studies; the use of a 2-way repeated measures ANOVA with groups (yoga, control) and time (baseline and posttreatment) would have been more appropriate than conducting both independent and paired *t*-tests given the study designs used [28–31]. On the other hand, one study [32] used *t*-tests to evaluate pre- and postdifferences both within and between groups but verified some findings by means of an ANOVA and another study employed a repeated measures ANOVA with Bonferroni-corrected comparisons, appropriate to the study design [33]. Primary outcome measures included maternal-related variables at various time points throughout pregnancy, during labour, and immediately after birth as well as infant-related variables at birth. 

Scores on each item of the Jadad scale and Delphi List are illustrated in Tables 3 and 4, respectively. Overall, Jadad scores ranged from zero to five; two studies were of high quality, scoring a five on the scale and four were of low quality, scoring a zero, one, or two on the scale. The more detailed Delphi List resulted in slightly greater variability in scores, which ranged 3–6 out of 9 possible points. Not one study was awarded points for care provider or participant blinding.

## 4. Description of RCTs and Controlled Studies Evaluating Yoga for Pregnancy

Rakhshani et al. [31] evaluated the effects of integrated yoga on the quality of life and interpersonal relationships of 102 healthy pregnant women when compared to standard antenatal exercises, using an RCT design. The 16-week-long integrated yoga program went from the 20th to 36th weeks of gestation and included lectures, breathing exercises (*pranayama*), physical postures (*asana*), meditation (*dhyana*), and a deep relaxation technique. The yoga group showed significantly greater improvements than the control group on the physical, psychological, environmental, and social domains of the World Health Organization Quality of Life Inventory (WHOQOL-100) as well as on the Expressed Inclusion and Wanted Control facets of the Fundamental Interpersonal Relationships Orientation (FIRO-B) questionnaire. Strengths of the study include an RCT design and a large sample size. The authors suggest that yoga is a noninvasive and cost-effective way of improving quality of life and interpersonal relationships during pregnancy.

Satyapriya et al. [32] used an RCT design to compare the effects of a similar 16–18-week-long integrated yoga program (plus a chanting component) with a control group that received standard prenatal exercises on maternal stress during pregnancy. Women who participated in the program were recruited between the 18th and 20th week of pregnancy and participated until their 36th week. The self-report perceived stress scale (PSS) and objective measures of heart rate variability (HRV) were used to measure stress. Heart rate was measured continuously before, during and after a deep relaxation technique (DRT) period in the yoga condition and of a corresponding supine rest (SR) period in the control condition. Three measures of heart rate were collected for analysis of continuous heart rate recording: low frequency (LF: related to sympathetic modulation), high frequency (HF: related to efferent vagal activity), and the ratio of low frequency to high frequency (LF/HF: related to sympathovagal balance).

Pre- to postintervention comparisons within each group showed that PSS scores decreased significantly in the yoga group and increased significantly in the control group so that by the end of the intervention PSS scores were significantly lower in the yoga group compared with the control group. Significant decreases in LF heart rate and the LF/HF ratio and significant increases in HF heart rate were observed from before to during the DRT and SR periods of the yoga and control conditions at the 20th and 36th weeks of gestation. At the 36th week, LF heart rate decreased from before to after in only the DRT (yoga) practice, whereas a significant before-to-after increase on HF and decrease on LF/HF ratio were observed in both groups. The results suggest that the DRT of the yoga condition may be a more powerful modulator of the sympathetic nervous system or the “fight or flight” response than the SR component of the exercise condition. Strengths of this study include an RCT design, a large sample size, an objective physiological measure, and information on the reliability of the PSS for an Indian population. 

In addition to evaluating the effects of yoga on maternal experiences throughout pregnancy, Sun et al. [28] also examined the effects of a 12–14 week yoga program during weeks 26th–28th to 38th–40th weeks on pregnancy-related discomfort as measured by the Discomforts of Pregnancy Questionnaire (DoPQ) and maternal childbirth self-efficacy during labour/delivery, when compared to standard hospital care using a nonrandomized, controlled trial design. Although there were no differences between the two groups in discomfort between the 26th and 28th week of gestation, the yoga group reported significantly less discomfort in the 38–40th week of gestation period. Furthermore, women in the yoga condition had significantly higher self-efficacy expectancy and outcome expectancy in both the active and second stages of labour than the women in the control group, as measured by the Childbirth Self-Efficacy Inventory (CBSEI).

The majority of women (77.8%) in the yoga group did not experience any contractions while practicing yoga, very few (4.4%) experienced a contraction once every 10 minutes, and about a fifth (17.8%) experienced a contraction once every 30 minutes. The authors conclude that a prenatal yoga program is safe for pregnant women and can reduce the discomforts of pregnancy and increase maternal self-efficacy and self-confidence, but an RCT design is needed to confirm these findings.

Although Sun et al. [28] state that the study was double blind, the absence of information on who was blinded and during what phase of the intervention raise questions about how double blinding was possible. Other methodological problems include a nonrandom method of allocating participants to treatment groups and the use of a nonvalidated questionnaire (DoPQ) developed by study investigators to measure discomfort during pregnancy. Furthermore, the principal investigator, who was not a certified yoga teacher, taught the yoga intervention to participants at the initial practice session, which may have compromised the quality of the yoga program and may also have introduced experimenter bias. However, assets of this study are that it employs a highly reliable measure with unidimensional subscales (CBSEI) and includes reports on adverse effects.

The effects of a 10–12 week prenatal yoga program during weeks 26th–28th to 37th-38th of gestation on labour outcomes were also evaluated by Chuntharapat et al. [33]. Labour variables such as maternal comfort, self-reported and experimenter-observed pain, length of labour, augmentation, and use of medication as well as birth outcomes, such as Apgar scores, were assessed in an RCT comparing an integrated yoga program to routine nursing care. It was found that the yoga program resulted in significantly higher maternal comfort at three different assessment points during labour as well as at a 2 hr assessment point postlabour. Both self-reported and observed labour pain scores were significantly lower in the experimental group than in the control group, although, not surprisingly, pain scores did increase over time in both groups. Furthermore, results demonstrated that the first stage of labour and the total duration of labour were significantly shorter in women who had received the yoga intervention. Significant between group differences were not found in the number of participants in whom labor was induced, the use of pethidine or in the newborns' Apgar scores. Strengths include clear and detailed charts depicting the results and the use of a variety of instruments to evaluate pain. A potential limitation is that participants were allowed to practice more than three times a week and so the dose-dependent relationship between amount of time practicing yoga and the observed effects is unclear. The study researchers did provide information on internal consistency for some measures (VASTC, MCQ, and VASPS) and a reliability coefficient for the PBOS but did not include external reports of validity.

In a nonrandomized, controlled trial, Narendran, Nagarathna, Narendran, Gunasheela, and Nagendra [30] compared the effects of an ~20-week-long integrated yoga condition to a walking condition during weeks 18–20 of gestation until delivery, on pregnancy outcomes. Main outcome measures included birth weight and gestational age at delivery. Secondary outcomes assessed were pregnancy-induced hypertension (PIH), intrauterine growth retardation (IUGR), pregnancy-induced hypertension (PIH) with IUGR, duration of labour, mode of delivery, preterm delivery, and intrauterine death (IUD). The number of infants weighing over 2500 g was significantly greater for women who had participated in the yoga condition; however, the mean birth weight of infants did not statistically differ between the two groups. In addition, the number of women who experienced preterm labour (i.e., before 37 weeks) was significantly lower and complications such as IUGR and PIH with associated IUGR occurred significantly less often in the yoga group. The statistical analysis was inappropriate: assumptions of normality were not assessed and *t*-tests were conducted to analyze continuous variables, rather than a two-way repeated measures (group × time) ANOVA. The authors did not report the numeric values of the *t*-tests and chi-square tests, nor did they report any measures of variability for the relevant outcome variables. Moreover, this study was not randomized and contains a participant self-selection bias. Notwithstanding these problems, a strength of this study is that it did not exclude women with medical conditions associated with pregnancy, such as gestational diabetes, or hypertension, making the results generalizable. 

In a publication stemming from the aforementioned trial [30], Narendran et al. [29] evaluated the same outcomes in women who were specifically selected as having abnormal Doppler readings of umbilical and uterine arteries. In this subsample of women, the authors reported that infants who were born to mothers in the yoga condition weighed significantly more and that a greater number of them weighed at least 2500 g when compared to the control group. By contrast, there were only trends in favour of the yoga group for pregnancy related complications and number of preterm deliveries. The study authors used the same statistical analysis as in the above trial and so problems resulting from an over-liberal approach are present for this study as well, and given the nonrandomized design, the results of this study are not definitive. Since abnormal readings of Doppler scores are associated with IUGR, this trial provides information about the safety of a yoga intervention for a high-risk population. Both publications [29, 30] from this trial reported that adverse effects did not occur in the yoga group.

## 5. Discussion

The purpose of the present paper was to evaluate evidence from controlled trials regarding the effects of yoga on the maternal experience during pregnancy and in labour as well as on birth outcomes. Overall, the results suggest that yoga is well indicated for pregnant women at a time in their lives when their hormonal, muscular, and psychological functioning undergo rapid change. The reviewed trials all use an integrated prenatal yoga program that spanned 10–20 weeks and all studies found improvements on a minimum of 1 outcome variable. Each study used at least two components of a yoga practice, postures, and meditation (*asana* and *dharana*), and the majority of the studies employed a gentle and integrated approach to Hatha yoga that also included breathing exercises (*pranayama*), lectures, chanting, and deep relaxation.

Notably, only three of the studies used a randomized, controlled design, and of those that did, only two described the randomization process; accordingly, only two studies received the maximum two points for randomization on the Jadad scale. It is difficult to double blind a yoga intervention and several of the included studies explained valid reasons why the double-blinding process was not appropriate for the design of their study. It is recognized that RCTs impose some logistical disadvantages, such as matters of cost, time, and geographical accessibility. In particular, Narendran et al. [30] would have compromised the large sample size if an RCT design had been used, given that women were travelling from neighbouring regions and may have dropped out if they had been assigned to a less accessible condition. Although care was taken to prevent between-group contamination [31, 32], some authors reported that they could not entirely prevent this, which may have confounded the results in that participants in the control condition may have utilized aspects of the yoga program (e.g., yogic theory or breathing exercises) [32] and others reported potential contamination bias from expectancy effects [28]. 

Three studies documented the presence or absence of adverse effects of the yoga intervention [28–30]; of these, two reported that there were no adverse effects observed. Information on rates of uterine contractions or other possible adverse effects of yoga during pregnancy, combined with details on the type of intervention used, would be informative for researchers designing future interventions. Early adverse events during pregnancy have been shown to have fetal neurobehavioural developmental consequences, so safety of the mother and infant during exercise-related activities is imperative [34]. Despite the general recommendations for physical exercise during pregnancy, there are still possible negative consequences, such as uterine contractions, maternal-fetal transfer of catecholamines, decrease of oxygen, premature labour, and nutrient flow or attenuation/decrease of fetal heart rate [9]. Guidelines have been proposed to ensure adequate management for the safety of the mother and fetus in exercise and related activities [9]. Various forms of exercise (e.g., aerobic and weight bearing) have different consequences on maternal and fetal physiology, especially across trimesters. For example, during the third trimester, both the mother and fetus are more vulnerable to physical stress [35]. With the exception of one study [28], most of the yoga programs evaluated in the present paper did not place an emphasis on “yoga as exercise” per se, and so are unlikely to be susceptible to the risks of more rigorous exercise regimes. Empirical evidence is needed to create guidelines outlining postures that are safe for pregnant women across trimesters. Regardless of the type of yoga or specific postures used, modifications should be made according to the specific needs of the individual woman, in the prevention of overexertion, stress on the fetus, and premature labour. Yoga is a low impact, easily modifiable and mindful activity, considering it is a safe and sustainable activity for pregnant women.

Although the studies reviewed in the present paper contribute valuable information about the potential effects of yoga on pregnancy outcomes, several limitations were noted. Firstly, four of the six studies that were included in this paper used an overly-liberal statistical approach, which should be considered in the interpretation of the effects of yoga for the corresponding outcome measures, including quality of life, interpersonal relationships, discomfort, self-efficacy, and birth outcomes [28–31]. Of the three studies that employed an RCT design, only two used an appropriate statistical analysis and so the results from these studies concerning stress, heart rate variability, comfort, pain, and length of labour are the most definitive [32, 33]. A second limitation observed is that many of the studies relied on a home-based yoga practice for logistical reasons, and, therefore, depended on self-report diaries and phone calls to assess adherence [28, 31–33]. None of the studies that monitored compliance reported any results on frequency or engagement of participants' yoga practice and only two listed this methodology as a limitation [31, 33]. 

A third limitation is that the use of a prenatal yoga program was primarily evaluated in populations of Asian nationalities (Taiwanese, Indian, and Thai); research is needed to evaluate the efficacy of yoga for pregnancy in populations from other continents. Cultural and demographic variables should also be considered in the interpretation of some results; it has been suggested that low birth weight and premature birth are persistent problems with no current solution and that prenatal yoga programs may prevent poor outcomes for these factors [29, 30]. Cultural context and health related issues of infant weight should be considered in the interpretation of the effects of a yoga program on infant weight. Lastly, the majority of the women in the reviewed studies were of middle-to-high socioeconomic status, presenting a selection bias of participants [28, 32, 33], thus reducing generalizability.

## 6. Future Directions

### 6.1. Methodological Improvements

In light of the limitations of existing research, we recommend several methodological improvements for future studies, including a more rigorous statistical approach when evaluating multiple outcome measures, use of more intensive self-report measures to monitor adherence such as interactive web-based monitoring systems, and inclusion of more diverse samples in terms of ethnicity and socioeconomic status. Future studies evaluating the effects of a yoga intervention on pregnancy-related outcomes should strive to use an RCT design, and, where possible in the research protocol, use methodology to prevent chance outcomes, allocation biases, and both researcher and participant expectancy effects. There is also a need to evaluate the efficacy of yoga for high-risk pregnancy populations, such as women with pregnancy-induced hypertension or diabetes and for women over the age of 35 years.

### 6.2. Dismantling Approach: The Challenge of Identifying the Critical Ingredients of Yoga

The present paper evaluated the components of the various yoga interventions (postures, breathing exercises, meditation, deep relaxation, anatomy, lectures, and chanting), which provides useful information on the quality, depth, and scope of each program. Given that the term “yoga” is broad and may denote any combination of the aforementioned components, it is important to operationally define what constitutes a yoga program in order to discern what is being evaluated. There is a debate in the field regarding the utility of a dismantling approach, as it does not acknowledge that yoga is inherently a holistic health practice and that such an approach will fail to capture its essential features or core mechanisms. This conflict reflects the different paradigms of yoga and science and their emphasis on holism and reductionism, respectively. 

From a research perspective, there is interest in better understanding which of the components are responsible for the observed effects and to uncover their putative mechanisms. It is possible that a dismantling approach, if done appropriately, may have theoretical and clinical value in that it may provide important information about the specific components that lead to particular psychological and physiological effects. On the other hand, dismantling yoga into various components presents both theoretical and practical challenges. When dismantled into specific components, it can be argued that the practice is “yoga-like” but not yoga, and that the beneficial effects accrue only when yoga is practiced as a holistic entity, not unlike the experience of musical harmony that emerges from a barbershop quartet and disappears if one was to listen separately to each of the four parts. Nevertheless, this is an empirical question that can be tested by ensuring that any dismantling study incorporates as a control group a true yoga condition. Another challenge of the dismantling approach is the logistical difficulty of matching interventions based on the various elements of yoga; classes based upon meditation or chanting may differ in length from classes of asana, making them difficult to compare. Finally, there is a risk for the dismantling approach to be conducted ad infinitum, in which types or sequences of asanas or forms of pranayama may be compared. It may be most effective, from a clinical perspective, to compare yoga interventions to other commonly used approaches to treatment, such as aerobic exercise, pharmacological management, or other mind-body practices such as Tai Chi and Qi-Gong.

### 6.3. Mindfulness and Other Mind-Body Interventions

An active ingredient in a yoga program may be mindfulness, which has been effective in symptom reduction and general health improvement in a variety of conditions that are relevant to pregnancy, such as anxiety, depression, back pain, and stress [36]. Moreover, preliminary research from a mindfulness-based childbirth and parenting education adaptation of a traditional mindfulness-based Stress reduction program found improvements in measures of anxiety, depression, and positive affect in women participating in their third trimester of pregnancy [37]. Similarly, an RCT evaluating a psychosocial mindfulness-based intervention administered in the second half of pregnancy found reductions in anxiety and negative mood when compared to waitlist control, indicating mindfulness-based interventions are a possible mental health approach to managing stressors associated with pregnancy [38]. The evaluation of mindfulness and endocrine, immune, or neurological variables in an integrated prenatal yoga program or in dismantled components may provide valuable insight regarding the key components responsible for generating change. In addition to evaluating mindfulness as a construct within a yoga intervention, we also recommend that future research compare the effects of a prenatal yoga program with the effects of traditional mindfulness-based stress reduction programs, mindfulness based therapies, and other mind-body practices, such as tai chi, on maternal and infant prenatal outcomes.

### 6.4. Effects of a Prenatal Yoga Program on Pre- and Postnatal Maternal and Fetal Variables

The reviewed studies provide empirical support for the efficacy of integrative yoga programs for pregnant women, but we know little about how the specific components of yoga may impact maternal pain, physiological, and psychosocial variables as well as fetal or infant parameters. A dismantling design may provide valuable information regarding the ways that different components of yoga may alter maternal nervous system functioning and in turn influence fetal neurophysiology or behaviour. For instance, paced breathing exercises, which might be comparable to a yogic breath practice (*pranayama*), have been shown to be associated with acute changes in fetal heart rate in response to uterine stimulation [39]. In addition, fetuses of mothers who had received an intervention consisting of relaxation techniques, such as progressive muscle relaxation and guided imagery, had higher long-term heart rate variability than controls, and women who had received progressive muscle relaxation had significantly more uterine activity than the guided imagery or control groups [8]. It is possible that elements commonly included in a yoga practice, such as breathing exercises or deep relaxation, may affect both fetal heart rate and fetal movement. We recommend evaluating the effects on the fetus of maternal breathing exercises or deep relaxation when done as part of a comprehensive yoga program.

Pregnancy can be a stressful time for expectant mothers, and it has been suggested that pregnancy associated stress can have adverse effects on fetal development during critical periods, resulting in poor outcomes for length of gestation, fetal growth, birth weight, fetal development, and general programming of the nervous system [5, 8]. For instance, elevated levels of maternal cortisol, a stress hormone, in the second and third trimester of pregnancy are associated with an increased response of infant cortisol to a heel-prick procedure after birth [40]. These results point to the importance of evaluating the effects of a prenatal yoga intervention on the relationships between (1) maternal hypothalamic-pituitary-adrenal axis and sympathomedullary pathway and (2) changes in stress levels of the fetus by measuring variety of stress related maternal (e.g., cortisol, heart rate, and self-reported measures) and fetal (e.g., activity level and heart rate) variables over the course of pregnancy and in the early postpartum period.

It is possible that alteration of maternal sympathetic nervous system functioning, as demonstrated by reduced levels of stress-related hormones such as cortisol, may be one of the mechanisms through which yoga initiates psychophysiological change in pregnant women. An integrated yoga program, including *asana*, *pranayama*, and *dharana*, guided relaxation, and yogic theory in application to pain states, has been shown to alter cortisol levels in a sample of females with chronic pain due to fibromyalgia [41]. Yoga has also been shown to reduce inflammatory markers, decrease heart rate, and produce improvements in physical fitness variables, all of which may work in concert with behavioural change and psychosocial functioning to improve reactivity to stress and pain [2]. 

Our search strategy did not yield a single published paper examining how a prenatal yoga program affects maternal adjustment to demands in the postnatal period, such as breast feeding, physical healing and recovery from birth, and sleep deprivation, amongst others. Follow up evaluation of these variables or other stress-related measures such as cortisol, at one month or six months after birth may provide insight into the lasting impact of a yoga program. It would also be useful to evaluate stress, pain, cognition, and physiological variables in infants at follow-up time periods in order to better understand the implications of a prenatal yoga program on development in certain domains during the early years of life. In addition, yoga interventions in the postpartum period may be effective in addressing these areas of concern as well as in the treatment or prevention of specific maternal conditions associated with this time period, such as postpartum depression.

## 7. Summary

Given the specific physical needs of women during pregnancy, a tailored and specialized yoga protocol that uses a variety of elements of a yoga practice is best indicated. Several of the reviewed studies provide a holistic approach to health promotion and stress management, providing participants with a framework with which to integrate the lecture material on yogic philosophy, positive lifestyle change, mindful awareness, stress reduction, and pregnancy and labour into their daily lives. It is recommended that future research studies use yoga interventions that fall under the general category of Hatha yoga or use programs in line with a particular school of yoga that emphasizes a specialized, gentle and modified *asana* programs, such as Iyengar or restorative yoga. Research-based interventions should not use types of yoga that emphasize a physical demanding, strength-based, or heated practice for safety precautions for both the mother and fetus. 

The present paper has several limitations. Because of the sparse number of RCTs and the absence of double blind RCTs in the literature, we included studies that were randomized but not double-blinded and also controlled trials that lacked randomization. Due to the relatively few articles included in the paper, the findings outlined are preliminary and not conclusive or generalizable. 

## 8. Conclusions

In conclusion, the present paper suggests that a prenatal yoga program results in benefits during pregnancy as well as throughout labour and on birth outcomes. This budding body of work suggests that improvements were observed on psychological domains during pregnancy and labour (e.g., quality of life and self-efficacy), on physical and pain measures during labour (e.g., discomfort and pain), and on birth variables (e.g., birth weight and number of preterm births). The only adverse health outcome that was reported was uterine contractions, which can be monitored with a modified approach and appropriate activity reduction. Overall, the evidence that yoga is well suited to pregnancy is positive, but methodological problems with the published literature and a general insufficient wealth of published trials make it impossible to draw any firm conclusion. Our recommendations above will allow researchers to work alongside yoga practitioners to craft potent, standardized programs that are also amenable to evidence-based evaluation in a research environment.

## Figures and Tables

**Table 1:** Controlled studies of yoga for pregnancy.

First author, year	Study design	Participants (pregnant women)	Experimental intervention	Control intervention	Outcome measures	Main results
Rakhshani, 2010 [31]	Prospective, two-armed RCT	18th–20th week of gestation, 20–35 yrs of age, PG or MG with at least one living child (*N* = 111, *n* = 102)	Integrated yoga, 20th–36th week of gestation. First month of yoga was done with instructor, following months done at home (1 hour, 3 times a week). Refresher classes were provided at each antenatal visit.	Standard antenatal exercises, 20th–36th week gestation, same format as the yoga intervention.	WHOQOL-100, FIRO-B	Significant improvements in the yoga condition for physical, psychological, environmental, and social domains of the WHOQOL-100 and on expressed inclusion and wanted control of the FIRO-B when compared to the control group.

Satyapriya, 2009 [32]	Prospective, two-armed RCT	18th–20th week of gestation, 20–35 years of age, PG or MG with at least one living child (*N* = 122, *n* = 90)	Integrated yoga and yogic relaxation, 20th–36th week of gestation (until delivery). First month of yoga was done with instructor (2 hours, 3 times a week), following months done at home with cassette (1 hour, 3 times a week). Refresher classes were provided 1 time a month until week 28 and 2 times a month until week 36.	Standard prenatal exercises, 20th–36th week gestation, same format as the yoga intervention.	PSS, HRV	The yoga group's stress scores decreased, while the control group's stress scores increased. Improved autonomic response in both yoga and control group.

Chuntharapat, 2008 [33]	RCT	26th–28th weeks of gestation, ≥18 years of age, PG (*n* = 74)	6 × 1 hour yoga classes on weeks 26th–28th, 30th, 32nd, 34th, 36th, and 37th week of gestation as well as 1 hour practice at home, at least 3 times a week after the first class for 10–12 weeks.	Routine nursing care, including casual conversations for 20–30 min, during hospital visits.	STAI pretrial, VASTC, MCQ, VAPS, PBOS, Apgar scores, length of labor, birth augmentation, use of pethidine	Experimental group demonstrated significantly higher levels of comfort, lower levels of self-reported, and experimenter observed pain throughout labour and shorter duration of labour than the control group.

Sun, 2010 [28]	Nonrandomized controlled trial	26th–28th weeks of gestation, ≥18 years, PG (*N* = 96, *n* = 88)	Initial yoga class was taught by investigator, followed by home practice video of 30 min, 3 times a week for 12–14 weeks.	Standard hospital care.	DoPQ, CBSEI	Decreased pregnancy discomfort and increased childbirth self-efficacy in the yoga group when compared to standard care.

Narendran, 2005 [30]	Prospective, matched, and observational study	18th–20th weeks of gestation, 18–35 years of age, PG or MG (*n* = 335)	Yoga practice 1 hour daily until delivery, taught in the first week and refresher classes every 3-4 weeks.	Walking 30 min, twice daily from enrollment until delivery.	Birth weight, gestational age at delivery, PIH, IUGR, PIH with IUGR, duration of labor, mode of delivery, preterm delivery, IUD.	The number of infants weighing ≥2500 g was greater in the yoga group. The yoga intervention group presented with lower duration of labor and lower incidence of IUGR and PIH with IUGR than the control group.

Narendran, 2005 [29]	Prospective, matched, and observational study	18th–20th weeks of gestation, 18–35 years of age, PG or MG, women who had abnormal Doppler readings of umbilical and uterine arteries (*n* = 121)	Yoga practice 1 hour daily until delivery, taught in the first week and reviewed every 3-4 weeks with instructor.	Walking 30 min, twice daily from enrollment until delivery.	Birth weight, gestational age at delivery, PIH, IUGR, PIH with IUGR, duration of labor, mode of delivery, preterm delivery, IUD.	Greater number of infants weighing ≥2500 g was higher in the yoga group.

WG: weeks of gestation, PG: primigravida, MG: multigravida, WHOQOL-100: World Health Organization Quality of Life Inventory-100, FIRO-B: fundamental interpersonal relationships orientation-B, PSS: perceived stress scale, HRV: heart rate variability, STAI: state-trait anxiety inventory, VASTC: visual analogue scale of total comfort, MCQ: maternal comfort questionnaire, VAPS: visual analogue pain scale, PBOS: pain behavioural observation scale, DoPQ: discomforts of childbirth questionnaire, CBSEI: childbirth self-efficacy inventory, PIH: pregnancy-induced hypertension, IUGR: intrauterine growth retardation, IUD: intrauterine death.

**Table 2:** Components of yoga intervention for each study.

Author (year)	Postures (*asana*)	Breathing exercises (*pranayama*)	Concentration/meditation (*dharana*/*dhyana*)	Deep relaxation/yoga sleep (*nidra*)	Lecture/ counseling	Anatomy	Chanting
Rakhshani et al. (2010) [31]	×	×	×	×	×		
Satyapriya et al. (2009) [32]	×	×	×	×	×		×
Sun et al. (2010) [28]	×		×				
Chuntharapat et al. (2008) [33]	×	×	×	×		×	×
Narendran et al. (2005) [30]	×	×	×	×			
Narendran et al. (2005) [29]	×	×	×	×			

**Table 3:** Score breakdown on the Jadad scale for each study.

Author (year)	Randomization and explanation	Single blinding and explanation	Description of participant withdrawal/dropout	Total score
Rakhshani et al. (2010) [31]	2	2	1	5
Satyapriya et al. (2009) [32]	2	2	1	5
Sun et al. (2010) [28]	0	0^∗^	1	1
Chuntharapat et al. (2008) [33]	2	0	0	2
Narendran et al. (2005) [30]	0	0	0	0
Narendran et al. (2005) [29]	0	0	0	0

^∗^Study authors state that the methodology was double blind, but in light of other information provided, it is clear that it was not.

**Table 4:** Score breakdown on the Delphi List for each study.

Author (year)	Randomized	Treatment allocation concealed	Similar baseline characteristics	Eligibility criteria specified	Outcome assessor blinded	Treatment provider blinded	Patient blinded	Point estimates/ variability	Intention-to-treat analysis	Total
Rakhshani et al. (2010) [31]	1	0	1^∗^	1	1	0	0	1	0	5
Satyapriya et al. (2009) [32]	1	1	1^∗^	1	1	0	0	1	0	6
Sun et al. (2010) [28]	0	0	1	1	0	0	0	1	0	3
Chuntharapat et al. (2008) [33]	1	0	1	1	0	0	0	1	0	4
Narendran et al. (2005) [30]	0	0	1	1	0	0	0	0	1	3
Narendran et al. (2005) [29]	0	0	1	1	0	0	0	1	1	4

^∗^All baseline characteristics were matched except for professional status.
